# Hospital Accounts and Finance

**Published:** 1923-05

**Authors:** Joseph E. Stone

**Affiliations:** (Accountant, St. Thomas's Hospital, London)


### HOSPITAL ACCOUNTS AND FINANCE.

### GETTING VALUE FOR MONEY.

By JOSEPH E. STONE, F.S.A.A., F.S.S., F.R.Econ.Soc. (Accountant, St. Thomas's Hospital, London.)

FINANCIAL transactions are commonly considered in the light of their immediate effect, and often merely represent the exchange of cash into some other form of commodity or service. Very briefly, they may be divided into items of expenditure, items of income, and changes in the nature of existing assets and liabilities. These three classes of transactions are intimately bound up one with another and cannot always be considered separately, yet each is of equal importance; an example will make this quite clear. The sum of £100, either in the form of cash in hand or as a credit at the bank, is looked upon as a rather sacred thing, and members of Committees and executive officers would not think of dealing with it without going through some form of ritual. Let the £100 be turned into an asset of another kind—stores or a man's services—and the tendency is to regard it in a much more familiar manner, if not to ignore it altogether. From the financial point of view, however, control is as necessary for the correct utilisation of the stores or the man's services as it is for actual cash, and the same rules should apply as are so carefully observed in the case of the £100. This point cannot be over emphasised, and in the consideration of financial control it will be obvious that for the scheme to be complete and effective it must embrace every form of transaction.

### Unity of Control.

These transactions are carried out in various departments, and it is necessary that control should be extended to these departments, and that each should have in operation certain schemes or systems of control as affecting only the work of the department. To be effective, however, these various systems must be co-ordinated to form a complete whole, and as the transactions have to be recorded in books of account kept in the accountant's department it is clearly this officer's duty to see that the control is sufficient and effective. By financial control, then, is meant unity of financial control exercised by the accountant, who should act under the direct supervision of the Finance Committee.

### The Accountant's Functions.

It will readily be admitted that this unity of control is not reached merely by investing this officer with the powers indicated. He must be supported by appropriate regulations and a carefully considered scheme of organisation. The reason for this is obvious. If we consider for a moment the functions of a large general hospital we shall find that the accounts will differ fundamentally in scope and content from those of commercial undertakings. A hospital does not exist for profit-making; its accounts are at best only an instrument which may be used by some expert intelligence in conjunction with other factors as a basis of judgment. To take the place of the profit and loss account it is therefore necessary to substitute a parallel system of administration and financial control. The administrative officers are primarily concerned with the execution of their various departmental activities, and the accountant watches these services and activities coldly and critically from the point of view of cost. The accountant, with his financial organisation, is not intended to put a drag on the wheel of progress, but to ensure as far as possible that the hospital gets value for money, that there is no extravagance, that the most economical methods are adopted, and generally by criticism and financial vigilance to serve the same purpose as the profit and loss account in a commercial concern.

### Paramountcy of the Finance Committee.

It is difficult to imagine a hospital of any size which does not possess recognised rules for the control of its finances, yet there appears to be few cases where these rules are properly collated, codified, and issued for reference to all officials and members of committees. Oftentimes rules and regulations lose one half of their effectiveness when they are scattered about in a score of places among miscellaneous papers and minute books. It is suggested that hospitals should have in operation a complete set of Standing Orders concerning their finances, and copies should be issued to every official and every member of committees. They should be strong and complete enough to ensure the proper administration of all the financial transactions in every branch and department of the hospital activities. The following suggestions, which are by no means exhaustive, are put forward as being those which will prove beneficial :—

1. That the whole of the account keeping of the hospital should be centralised under the control of the accountant, who should be directly responsible to the Finance Committee.

2. That certian specified returns should be prepared and presented to this committee at stated intervals. These would include a statement of estimated annual income and expenditure; a periodical statement comparing actual with estimated income and expenditure, showing clearly cases of over spending and explanations of officers concerned; interim income and expenditure accounts on a comparative basis; and miscellaneous financial and statistical statements arranged in summarised form to show leading facts not included in the foregoing returns.

3. That the Finance Committee shall ration each spending department or committee up to an amount equivalent to their approved estimates.

4. That no excess expenditure over sanction be incurred without submission of a report on the necessity for such expenditure being made to the Finance Committee and their approval by resolution obtained.

5. That any liability to be incurred likely to exceed an agreed sum to be incurred only after estimates or quotations have been obtained and submitted for approval.

6. That purchases may not be made except on the authority of the Finance Committee made after submission of requisitions from the spending departments or committees.

7. That all orders for goods or work required to be done shall be on official forms, and that such orders shall emanate from the Secretary only.

8. That all contracts, tenders, &c., be opened by the Finance Committee in the first place.

9. That no expenditure of a capital nature by any committee or department be made until a full report has been made to the Finance Committee on the proposal and the cost involved, both initial and maintenance.

10. That all motions which may involve directly or indirectly expenditure not otherwise provided for or increased expenditure, or which would interfere with the financial policy of the hospital, should stand referred to the Finance Committee for consideration and report to a Grand Committee or Court before any further steps are taken.

### Necessity for a Strong Finance Committee.

Every hospital of sufficient size ought to appoint a Finance Committee with full powers of control over all matters relating to money, and they ought to have the absolute right to exercise such powers, subject, of course, to the confirmation of a Council, Grand Committee, or Court. It should be a Committee of such strength that though confirmation by a higher authority is necessary, its decisions would not be lightly disregarded. It is, or ought to be, the most important Committee of the hospital, and its constitution should be such as to command the full confidence of the governing body and the officials. This view of a Finance Committee coupled with its powers as expressed in the standing orders is at once opposed to the present popular but erroneous view so often heard, that "the Finance Committee is only the committee which passes the bills." A strong and efficient Finance Committee is a great asset to any hospital, and upon the careful and thorough manner in which its duties are carried out depends to a large extent successful and economical administration.

### A Flourishing Hospital.

The principle of the contribution by each hospital patient towards the general fund has been in existence at Cirencester Hospital since its foundation in 1875, and is considered by the committee to be one of the reasons for its popularity. The hospital has increased each year in usefulness, and has always paid its way. A considerable number of private paying patients are taken, and a large number of these either become regular subscribers or give donations or improvement in equipment. In 1920 a scheme was inaugurated by which the various firms in the town undertook to receive a weekly subscription of threepence from their employees. In many cases the employers add 100 or 50 per cent. to their employees' subscriptions, and this year £747 has been received from this source.

